# Clinical Quiz—A Rare Case of Anal Canal Duplication in the Context of Currarino Syndrome

**DOI:** 10.1055/s-0041-1735595

**Published:** 2021-11-10

**Authors:** Sean Jared Connor, Giulia Brisighelli, Nirav Patel, Marc A. Levitt

**Affiliations:** 1 Department of Paediatric Surgery, Chris Hani Baragwanath Hospital, Johannesburg, Gauteng, South Africa; 2 University of the Witwatersrand School of Clinical Medicine, Johannesburg, Gauteng, South Africa; 3 Department of Paediatric Surgery, University of the Witwatersrand, Johannesburg, Gauteng, South Africa; 4 Department of Surgery, Colorectal and Pelvic Reconstructive Surgery, Children's National Hospital, Washington, District of Columbia, United States

**Keywords:** currarino syndrome, anal canal, anorectal malformation, sacral defect, congenital abnormalities

## Abstract

Currarino syndrome (CS) is a rare condition that presents with any combination of a sacral defect, a presacral mass, and an anorectal malformation. This collection, referred to as Currarino's triad, may not necessarily present as all three abnormalities in the diagnosis of the syndrome. Anal canal duplication (ACD) is an even rarer occurrence. We present a case that lies on the CS spectrum with an associated ACD and discuss a complex surgical challenge that necessitated a customized management plan, devised through a multidisciplinary approach.

## Introduction

Currarino syndrome (CS) is an extremely rare collection of anatomical abnormalities. CS presents as an arrangement of a sacral defect, a presacral mass, and an anorectal malformation. 1 The large variation in expressivity of malformations, and subsequent wide-ranging clinical severities, makes CS both easily overlooked and difficult to manage.

CS was first described by the Italian-born, American radiologist, Guido Currarino. He was a pediatric radiologist at the Southwestern Medical School and Children's Medical Center, Texas, USA. Currarino first described the triad in a case series in 1981, in which he looked at radiological imaging of three children born with anorectal abnormalities. The definition later evolved to include cytogenetics as a causative factor. 1

Anal canal duplication (ACD) is a rare malformation in which there is a second perineal orifice, usually posterior to the native anus. The ACD may be blind ending or communicating with the anal canal. The two structures frequently share a common wall. 2 3 In some instances, a duplication on the sagittal plane has also been described with the two anuses parallel to each other and sharing the medial wall.

There is a paucity of literature describing CS and ACD as well as their management. To the best of our knowledge, our case represents only the fifth report in the literature, 3 4 5 6 which describes the coexistence of CS and ACD and the resultant unique surgical challenge. Of the 58 cases of ACD reported in the literature, we identified four patients who had an associated Currarino triad. Nine patients were not labeled as a CS but presented with presacral masses or sacral defect. 7 We believe genetic testing was never performed, and therefore these were never labeled as CS. We discuss possible surgical approaches with consideration of relevant and related clinical scenarios and potential complications.

## Case Report/ Images

**Fig. 1:**
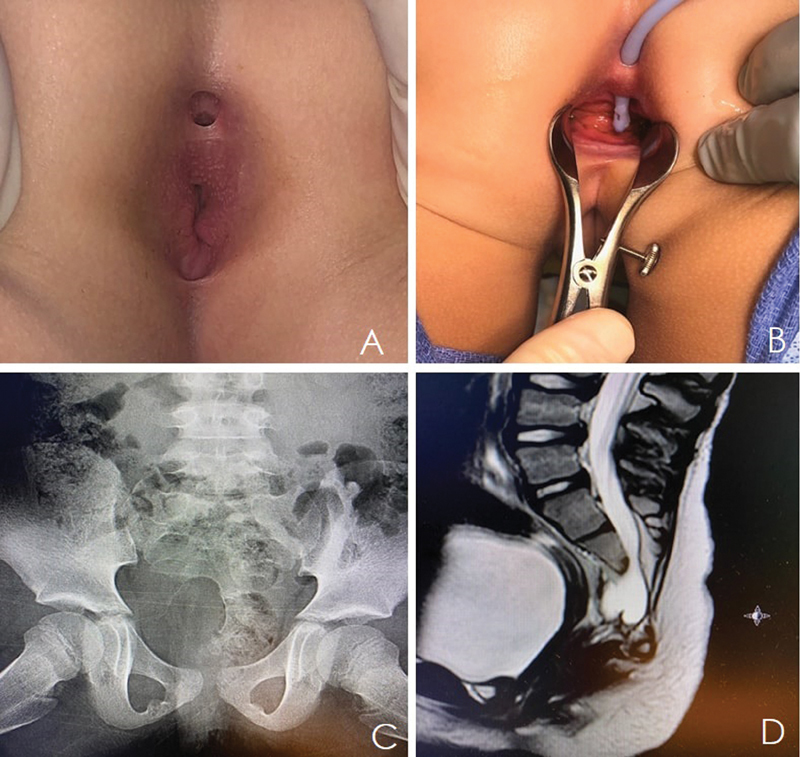
( **A, B** ) Anal canal duplication, communicating with the native anus. ( **C** ) Hemisacrum demonstrated on anteroposterior pelvic X-ray. ( **D** ) Abdominopelvic MRI showing a presacral mass on sagittal view.

A female patient presents with a perineal finding, which we suspected was a posterior anal duplication ( Fig. 1A, B ). The native anus, located anteriorly, appears to be of normal caliber and surrounded by the sphincter muscle complex. The abnormal orifice is located at the 6 o'clock position, posteriorly. When probed ( Fig. 1B ), it appeared to be communicating with anal canal distal to the dentate line and just proximal to the anal verge. Plain film anteroposterior pelvic X-ray shows a sacral abnormality ( Fig. 1C ). A magnetic resonance imaging (MRI) of the pelvis demonstrated a presacral mass consistent with an anterior meningocele and a conus at L2 but without spinal cord tethering ( Fig. 1D ). These features suggested that there is an ACD as part of a CS. Genetic testing was provided to the family, given the CS.

## Discussion

### Currarino Syndrome (CS)

The majority of patients diagnosed with CS are detected at birth because of the presence of an anorectal malformation (ARM). They undergo prescribed screening, including an AP of the sacrum and spinal ultrasound. Any type of ARM can be associated with CS, but most frequently it is anal stenosis or rectal atresia. Further investigations may reveal sacral anomalies (including a sickle-shaped sacrum [scimitar] or sacral agenesis), a presacral mass (sacrococcygeal teratoma, a meningocele or an enteric cyst), and/or spinal abnormalities such as tethered cord. 8 9 There is a familial tendency in approximately 70 to 90% of cases with an autosomal dominant pattern of inheritance and an identified defect in the HLXB9 gene on the 7q36 chromosome. It can, however, be sporadic in 10 to 30% of cases with no identifiable underlying genetic defect. 10 11 12 Anal stenosis can go undetected until the patient presents with constipation. In addition, once a stenosis is diagnosed, cases of dilations only without screening for a presacral mass have occurred, leading potentially to missing the presence of a presacral teratoma.

### Anal Canal Duplication (ACD)

Slightly over 90 cases of posterior anal canal duplications have been reported. Females are predominantly affected with a 1:11 male to female ratio. 2 3 Over half of patients are diagnosed before the age of 1 year, and the severity of symptoms tends to increase in correlation with the age at presentation. 13 Often an incidental finding, the subtlety of the malformation, and the overall rarity has resulted in ACDs going undiagnosed until adulthood, usually as a result of local complications such as constipation, anal pain or discharge, perineal/perianal infections and abscesses. ACDs are often mistaken for fistula-in-ano when presenting as an adult. 2 3 The etiology of ACD is unknown, although they are thought to arise from a duplicated dorsal cloaca. An alternate theory is that the rupture of the cloacal membrane and subsequent recanalization results in the formation of an anomalous anal canal. 13 The clinical workup of a patient with ACD includes a thorough clinical examination to identify any associated abnormalities. Associated abnormalities may be present in up to 44% of cases and include craniofacial (cleft lips), spinal (spina bifida), cardiac and renal defects. Midline defects are the most common association. 3 Additional investigations may include a fistulogram and a contrast enema to establish whether the orifice communicates with the native anal canal. 3 A literature review by Carpentier et al revealed that only 1 in 10 ACDs communicate with the anal canal and that the majority (74%) are tubular duplications with a minority (14%) being cystic duplications. 2 Surgical excision is advocated to avoid complications such as local sepsis and long-term risk of malignant transformation. 2 The mean age at which patients present due to complications is 34 months. 13 This supports the rationale for early diagnosis and excision.

Confirmative diagnosis of an ACD is made on histological examination of the resected duplicated tissue. Squamous epithelium can be found with areas of transitional and columnar epithelium. The excised tissue may contain anal glands and be surrounded by smooth muscle. 13

### Currarino Syndrome Plus Anal Canal Duplication

There is no clear explanation as to why posterior ACD occur in the context of CS, although given the reported association between CS and ACD, when an ACD is diagnosed, CS should be suspected and investigated. An ACD can, however, occur in isolation or as part of a caudal duplication syndrome where the duplicated anuses are in a sagittal orientation to one another. A plain film pelvic X-ray should therefore be performed to exclude any sacral malformations. In patients younger than 6 months of age, an ultrasound of the spine and abdomen/pelvis may be performed to investigate for spinal anomalies and presacral masses. If the patient has a suggestive spinal ultrasound, or if the patient presents after 6 months of age, an MRI of the spine is indicated. It is important to note the presence of a tethered cord, as this finding directly impacts surgical decision-making in terms of a primary repair versus a staged repair with combined neurosurgical intervention. As CS infers a genetic risk, genetic screening and counselling should be offered to siblings and immediate family members of patients confirmed to have CS. 10

Management of the combined pathologies of ACD and CS requires a multidisciplinary approach involving the pediatric surgeon, neurosurgeon, geneticist, pediatrician, and colorectal nursing staff. Owing to the variety of their presentations and the overall rarity of these conditions, the surgical management of ACDs and CS are far from established. Consequently, neither the timing of surgery nor the optimal surgical approach are well-defined. To avoid possible complications such as local sepsis and long-term risk of malignant transformation, intrinsically associated with the presence of an ACD, we advocate for surgical excision before the age of 1 year. 2 13

Described surgical techniques include a perineal approach, mucosectomy, and a posterior sagittal approach. 14 The perineal approach and the mucosal stripping may be adopted for short (< 30 mm) duplications without an associated presacral mass. 4 Excision using a posterior sagittal approach would be recommended in our case to remove the posterior duplication, preserving its common wall with the anus and to concomitantly address the anterior meningocele.

Another important consideration is whether to remove the duplication primarily or with the protection of a diverting colostomy. We believe that in the case of an isolated ACD, primary excision is a feasible and safe option. In our particular case, however, a three-staged approach may be beneficial. This is because the anterior meningocele could be repaired by the neurosurgeon at the same stage as the ACD excision, thus permitting excision of the presacral mass in a clean environment. Alternatively, the ACD should be managed separately from any work being done on the dura to avoid infection.

For the purposes of alignment of presenting features and management plans, the following scenarios have been illustrated. The scenarios fall on a spectrum from uncomplicated presentations to more potentially complicated presentations.

**Scenario 1** —An isolated ACD without a presacral mass. An uncomplicated isolated ACD is not emergent, and surgical excision can wait until an appropriate age/weight is achieved. In this scenario, excision of the ACD may be performed primarily with a posterior sagittal or a perineal approach. A mucosectomy/mucosal stripping may also be considered. A protective colostomy may not be necessary as there is no presacral mass to excise. The indications for excision include constipation, anal pain, or discharge and perineal/perianal infections.
**Scenario 2** —An ACD with a presacral mass (not communicating with the spine). Here, we have a slightly more complicated presentation. The resection of the ACD and presacral mass may be performed in the same setting with or without a protective stoma.
**Scenario 3** —In scenario 3, we are presented with an ACD and a presacral mass, that is, involving the spine with spinal cord tethering, as demonstrated at MRI. In this scenario, a possible approach would be to perform the resection of the presacral mass in the first operative setting and then perform a delayed resection of the ACD in a different operative setting. Delayed resection of the ACD is to possibly avoid complications such as meningitis secondary to an enterothecal fistula. The delay in excision of the ACD is provided, in that it is noncommunicating with the native anal canal, and the patient is asymptomatic. A two-staged approach may also negate the need for a protective colostomy.
**Scenario 4** —This scenario further covers the end of the spectrum of presentations. An anal stenosis/atresia in the context of CS. Here, a divided stoma should be considered as the initial stage, followed by the delayed resection of the presacral mass and repair of the ARM. In this scenario, the function of the stoma is to decompress the gastrointestinal system, allow for a clean neurosurgical field for the resection of the presacral mass, prevent local wound sepsis to the posterior sagittal incision made with the anal repair, as well as allow for anastomotic healing.

Our clinical case aligns with scenario 3. Our goal is to ultimately restore perineal anatomy and maintain functionality while limiting the complications of the resection. The decision to utilize a divided stoma would depend on the scenario faced, the experience of the surgeon, and the risk of infection. Furthermore, regardless of the scenario encountered, a neurosurgical opinion should be sought with regard to the excision of the presacral mass.

The communication between the ACD and the native anal canal will require careful division of the anal sphincter muscle complex posteriorly. Meticulous dissection between the ACD and native anal canal must be completed, keeping in mind that the ACD and the native anus may share a common wall. Perforation to the native anal canal is a possible risk. A protective colostomy should be considered in this case. Postoperative complications such as sepsis and anal strictures with associated constipation must be sought postop and during follow-up consultations.

Caregivers must be made aware that the outcomes in terms of fecal continence are mainly associated with the presence of sacral or spinal defects and that the resection of the ACD does not ensure continence. 15 Because of the sacral and spinal defects, a neurogenic bladder could also be present. Included in the preoperative counselling, there should be the possible need for bowel management programs as well as serial interventions to manage fecal and urinary continence issues.

## Conclusion

The association of ACD and CS is an extremely rare surgical dilemma, demanding the involvement of a multidisciplinary team. All patients with ACD must be investigated for CS. Several considerations inform surgical management. There are currently no guidelines regarding primary versus staged repair nor the timing of surgical intervention. Numerous patient and surgical factors underpin surgical decision-making.

## Notes

Insights and Importance for the Pediatric Surgeon

- In the event of an anal canal duplication, always investigate for associated anomalies, including Currarino syndrome.
- The presence of Curarrino syndrome in a patient with anal canal duplication worsens the outcomes in terms of bowel control. This necessitates the need for a tailored and multidisciplinary approach in the surgical management and follow-up of affected patients.
- A three-staged approach with a protective colostomy should be considered, especially in the patients who require concomitant spinal surgery.
- The use of a protective colostomy is highly dependent on number of factors and is ultimately the choice of the surgeon.

## References

[1] Currarino G Coln D Votteler T Triad of anorectal, sacral, and presacral anomalies AJR Am J Roentgenol 1981 137 02 395 398 6789651 10.2214/ajr.137.2.395

[2] Trecartin A C Peña A Lovell M Anal duplication: is surgery indicated? A report of three cases and review of the literature Pediatr Surg Int 2019 35 09 971 978 31256296 10.1007/s00383-019-04509-x

[3] Carpentier H Maizlin I Bliss D Anal canal duplication: case reviews and summary of the world literature Pediatr Surg Int 2009 25 10 911 916 19727768 10.1007/s00383-009-2474-z

[4] Koga H Okazaki T Kato Y Lane G J Yamataka A Anal canal duplication: experience at a single institution and literature review Pediatr Surg Int 2010 26 10 985 988 20668865 10.1007/s00383-010-2653-y

[5] Ochiai K Umeda T Murahashi O Sugitoh T Anal-canal duplication in a 6-year-old child Pediatr Surg Int 2002 18(2-3):195 197 11956798 10.1007/s003830100691

[6] Yatsuzuka S Okamatsu T Ishikawa M A case with Currarino's triad Jpn J Pediatr Surg 1986 18 1627 1638

[7] Palazon P Julia V Saura L Anal canal duplication and triplication: a rare entity with different presentations Pediatr Surg Int 2017 33 05 609 617 28255623 10.1007/s00383-017-4074-7

[8] Caro-Domínguez P Bass J Hurteau-Miller J currarino syndrome in a fetus, infant, child, and adolescent: spectrum of clinical presentations and imaging findings Can Assoc Radiol J 2017 68 01 90 95 27887934 10.1016/j.carj.2016.05.007

[9] Jacquier C Dobremez E Piolat C Dyon J F Nugues F Anal canal duplication in infants and children–a series of 6 cases Eur J Pediatr Surg 2001 11 03 186 191 11475116 10.1055/s-2001-15482

[10] Köchling J Karbasiyan M Reis A Spectrum of mutations and genotype-phenotype analysis in Currarino syndrome Eur J Hum Genet 2001 9 08 599 605 11528505 10.1038/sj.ejhg.5200683

[11] Muhammad Abdelhafez M Ashraf Hamed S Incomplete Currarino syndrome: case report and a brief review of literatureArchives of Clinical Gastroenterology 2020 (e-pub ahead of print)https://doi.org/10.17352/2455-2283.000070

[12] Tucker A M Morgenstern P Diaz D Neurosurgical management of Currarino syndrome: a case series and review of literature Surg Neurol Int 2019 10 70 31528408 10.25259/SNI-26-2019 PMC6744743

[13] Lisi G Illiceto M T Rossi C Broto J M Jil-Vernet J M Lelli Chiesa P Anal canal duplication: a retrospective analysis of 12 cases from two European pediatric surgical departments Pediatr Surg Int 2006 22 12 967 973 17061104 10.1007/s00383-006-1801-x

[14] Tiryaki T Şenel E Atayurt H Anal canal duplication in children: a new technique Pediatr Surg Int 2006 22 06 560 561 16538439 10.1007/s00383-006-1654-3

[15] Dewberry L Peña A Meyers M L Mirsky D M Bischoff A Differentiating presacral masses in anorectal malformations and isolated sacrococcygeal teratomas Pediatr Surg Int 2019 35 09 979 983 31256295 10.1007/s00383-019-04510-4

